# Berberine Alleviates *Shigella*-Induced Dysentery by Regulating Intestinal Barrier and Inflammatory Responses

**DOI:** 10.3390/ijms27021063

**Published:** 2026-01-21

**Authors:** Jinwen Ding, Yu Zhang, Xinyu Fan, Yanxing Han, Yuan Lin

**Affiliations:** State Key Laboratory of Bioactive Substance and Function of Natural Medicines, Institute of Materia Medica, Chinese Academy of Medical Sciences & Peking Union Medical College, Beijing 100050, China; dingjinwen@imm.ac.cn (J.D.);

**Keywords:** Berberine, *Shigella* infection, dysentery, intestinal barrier, inflammatory responses

## Abstract

Berberine (BBR), an isoquinoline alkaloid, has a long history of clinical use in treating dysentery. However, its precise mechanism has not been fully elucidated. This study aimed to investigate the intestinal protective mechanisms of BBR against *Shigella flexneri* (*S. flexneri*)-induced dysentery in mice. We found that BBR significantly upregulated the intestinal barrier proteins ZO-1, occludin, and E-cadherin, enhancing intestinal mucosal integrity to inhibit *S. flexneri* invasion. Moreover, BBR effectively attenuated M1 macrophage polarization and restored the Th1/Th17/Treg balance to reduce inflammatory injury upon *S. flexneri* infection. Specifically, BBR reduced both the populations of Th1 and Th17 cells and their production of inflammatory cytokines IFN-γ and IL-17A. Concurrently, it enhanced Treg cell populations and the secretion of anti-inflammatory cytokines IL-10 and TGF-β1. Additionally, the intestinal protective effect of BBR was further augmented by an increase in secretory IgA (sIgA). Collectively, our findings demonstrate that BBR protects against *S. flexneri*-induced dysentery by enhancing the intestinal barrier and inflammatory responses, providing support for its clinical use.

## 1. Introduction

Dysentery from *Shigella* infection is a global health burden characterized by bloody diarrhea, abdominal pain, and intestinal inflammation. It leads to approximately 190 million cases and 200,000 deaths annually, with *S. flexneri* being the predominant pathogen in China [1,2,3]. Although antibiotics remain the first-line treatment, their clinical application is substantially compromised by the rising prevalence of antimicrobial resistance [4,5]. This threat was recently underscored by the World Health Organization’s (WHO) reclassification of fluoroquinolone-resistant *Shigella* as a “high-priority pathogen” [6]. Therefore, there is an urgent need for novel therapeutic strategies against *Shigella* infections.

Berberine (BBR), a traditional Chinese medicine extracted from herbs such as *Rhizoma coptidis* and *Cortex Phellodendri*, possesses a range of pharmacological activities, including antimicrobial, anti-inflammatory, anti-oxidant, anti-atherosclerotic, antidiabetic, anti-tumor, and neuroprotective properties [7,8,9,10]. BBR is widely used in China for treating dysentery due to its safety, efficacy, and low tendency for drug resistance [11]. Although its antibacterial effect was the first to be discovered and applied clinically, its precise mechanisms of action remain unclear. Our previous work demonstrated that BBR exerts therapeutic efficacy against *Shigella*-induced dysentery by acting on both the pathogen and the host: it directly inhibits the bacterial division targeting FtsZ protein and suppresses host macrophage pyroptosis to reduce intestinal inflammation and tissue damage [12]. Apart from this, we sought to investigate whether BBR protects against *S. flexneri*-induced dysentery through the alternative mechanisms.

As we know, the intestinal barrier prevents the translocation of harmful substances and pathogens (*S. flexneri*) from gut lumen into the systemic circulation [13]. Among the intestinal barrier, the physical barrier is a monolayer of intestinal epithelial cells, sealed together by tight junctions. These junctions are composed of ZO-1, occludin, and the adhesion protein E-cadherin, which regulate intestinal permeability and maintain barrier integrity, thereby preventing systemic exposure to pathogens and toxins. The key pathogenic mechanism of *S. flexneri* is characterized by epithelial invasion and junction disruption.

Beyond the physical barrier, the intestinal mucosal immune system maintains gut homeostasis through Th1, Th17, and Treg cells. Th1 and Th17 cells secrete cytokines (e.g., IFN-γ, IL-2, and IL-17A) to prevent pathogen dissemination and gut microbiota balance [14,15], while Treg cells suppress excessive inflammation to maintain immune tolerance [16]. However, pathogen infection disrupts this balance by activating Th1/Th17 responses, and the excessive cytokines impair Treg function, leading to mucosal damage [17]. Additionally, secretory IgA (sIgA) prevents pathogen adherence to epithelial cells by binding to them and forming immune complexes [18]. Therefore, the synergy between these immune cells and sIgA is fundamental to maintaining intestinal immune regulation.

In this study, we aimed to investigate whether BBR protected mice from *S. flexneri*-induced dysentery by preserving the intestinal physical barrier and regulating intestinal inflammatory responses. This work may provide a theoretical basis for the application of BBR to treat *Shigella* infection and promote intestinal health.

## 2. Results

### 2.1. BBR Alleviated the Symptoms of S. flexneri-Induced Dysentery in Mice

**Figure 1:**
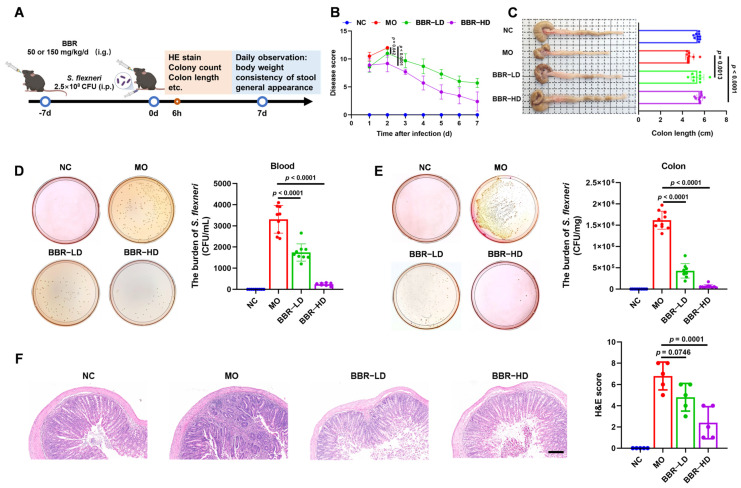
BBR alleviated the symptoms of *S. flexneri*-induced dysentery in mice. (**A**) Flow chart of the experiment. (**B**) Disease score. (**C**) Representative pictures of colon and the quantitative analysis of colon length (*n* = 9–10 mice per group). (**D**,**E**) *S. flexneri* counts in blood (**D**) and colon (**E**) (*n* = 8–10 mice per group). (**F**) Typical colonic histological sections stained with H&E (10 × magnification, scale bar = 100 μm) and statistics of H&E score (n = 5 mice per group). All data are presented as mean ± SD and compared with the MO group by one-way ANOVA with Tukey’s multiple comparisons. NC—normal mice with no treatment; MO—mice infected with *S. flexneri*; BBR-LD—mice treated with 50 mg/kg/d BBR; BBR-HD—mice treated with 150 mg/kg/d BBR.

To evaluate the therapeutic efficacy of BBR against dysentery, we utilized the established murine dysentery model induced by *S. flexneri* in our laboratory [12]. The experimental procedure is presented in Figure 1A. The disease scores were evaluated for seven days. Due to complete mortality in the MO group by the third day post-modeling, disease score was discontinued. Compared with the NC group, the MO group showed significantly elevated disease scores characterized by weight loss, stool consistency, and general appearance. Both BBR-LD and BBR-HD treatments effectively counteracted these effects, promoting weight gain and alleviating lethargy, piloerection, and diarrhea. Notably, the BBR-HD treatment exhibited a more pronounced efficacy in reducing disease scores (Figure 1B). Furthermore, quantification of colon length confirmed these therapeutic benefits, as both BBR-LD and BBR-HD treatments reversed the shortened colon in *S. flexneri*-infected mice (Figure 1C). Moreover, treatments with both BBR-LD and BBR-HD significantly reduced the bacterial load of *S. flexneri* in the blood compared to the MO group, as confirmed by the dilution plating on SS agar (Figure 1D). A similar significant reduction in *S. flexneri* colonization was observed in the colon tissues of the BBR-treated mice (Figure 1E). As shown in Figure 1F, H&E staining of the colon revealed BBR administration markedly ameliorated *S. flexneri*-induced pathological changes, including damaged epithelia, irregular crypt structures, and inflammatory cell infiltration. Quantitative analysis of the histological score showed that while BBR-LD had a limited effect, BBR-HD significantly reduced colonic injury, demonstrating the efficacy of BBR-HD treatment. Collectively, these findings suggested that the oral administration of BBR effectively mitigated *S. flexneri*-induced dysentery.

### 2.2. BBR Protected the Integrity of Intestinal Physical Barrier in S. flexneri-Induced Dysentery

**Figure 2:**
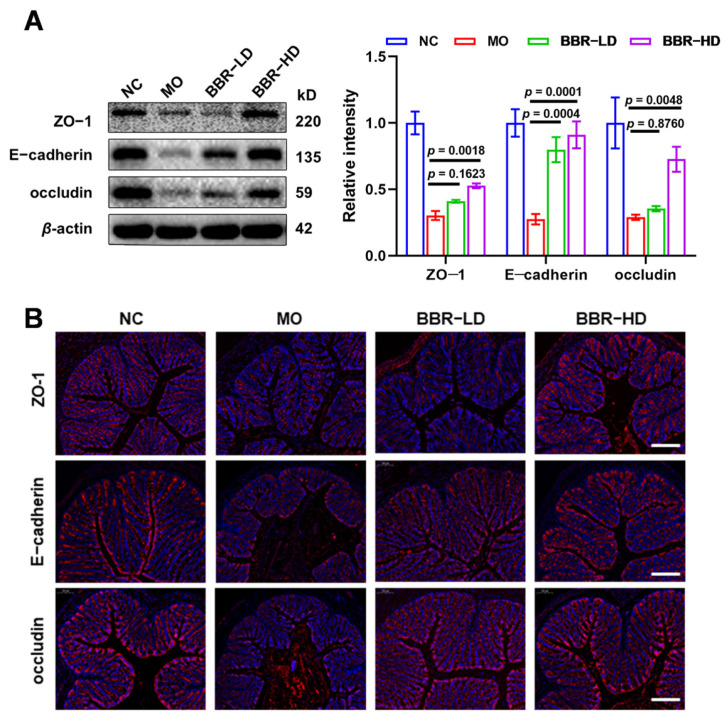
BBR protected the integrity of intestinal physical barrier in *S. flexneri*-induced dysentery. (**A**) Western blot assay of the protein levels of ZO-1, E-cadherin, and occludin (left) and quantitative analysis of protein expression (right). (**B**) Immunofluorescence staining was performed on colonic sections using antibodies against ZO-1, E-cadherin, and occludin (Scale bars = 100 μm). All data were based on values obtained from three replicates (n = 3 mice per group) and presented as mean ± SD and compared with the MO group by one-way ANOVA with Tukey’s multiple comparisons.

Given the critical role of intestinal barrier in maintaining mucosal homeostasis, we investigated whether the protective effects of BBR were mediated through the preservation of this barrier. The integrity of colonic epithelium, a core component of intestinal barrier, is maintained by tight junction proteins, including ZO-1, E-cadherin, and occludin. Therefore, to evaluate the impact of BBR on the intestinal physical barrier in *S. flexneri*-infected mice, we first examined the expression levels of ZO-1, E-cadherin, and occludin in colonic tissues by Western blot analysis. *S. flexneri* infection significantly decreased the expression of ZO-1, E-cadherin, and occludin in the colon. Conversely, BBR treatment upregulated the expression levels of these proteins, with BBR-HD exhibiting a more robust effect (Figure 2A). These observations were further identified using in situ fluorescent staining. Immunolabeling showed that the fluorescence intensity of ZO-1, E-cadherin, and occludin were notably reduced in the MO group. Remarkably, BBR treatment restored the staining patterns of these proteins, consistent with the Western blot results (Figure 2B). In summary, these results demonstrated that BBR, particularly at a high dose, effectively protected the intestinal physical barrier in *S. flexneri*-infected mice.

### 2.3. BBR Regulated the Intestinal Inflammatory Responses in Mice Infected with S. flexneri

**Figure 3:**
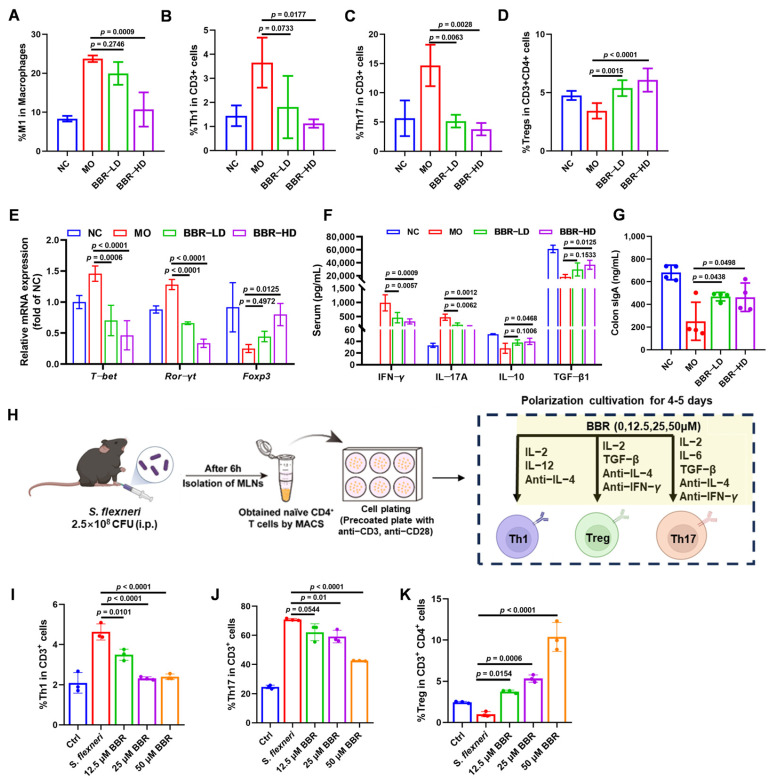
BBR regulated the intestinal inflammatory responses in mice infected with *S. flexneri*. (**A**–**D**) Proportions of M1 (**A**), Th1 (**B**), Th17 (**C**), and Treg (**D**) in MLNs of mice treated with or without BBR. (**E**) The mRNA levels of *T-bet*, *Ror-γt*, and *Foxp3* in the colon. (**F**) The quantitative analysis of serum IFN-γ, IL-17A, IL-10, and TGF-β1 by ELISA. (**G**) The expressions of colon sIgA. (**H**) Flow chart of the experiment. (**I**–**K**) Proportions of Th1 (**I**), Th17 (**J**), and Treg (**K**) in the in vitro polarization culture. Statistical analysis was performed using one-way ANOVA with Tukey’s multiple comparisons test. Data in panels (**A**–**G**) represented individual mice, with each group containing *n* = 3–4 mice; significance was indicated relative to the MO group. Data in panels (**I**–**K**) were representative replicates from three independent in vitro experiments; significance was indicated relative to the *S. flexneri* infection group. All data are presented as mean ± SD.

Intestinal immune cells and their secreted cytokines play a pivotal role in the rebalancing of inflammatory responses. We therefore investigated whether BBR regulated these cells to combat *S. flexneri* infection. Flow cytometric analysis of cells from murine mesenteric lymph nodes (MLNs) revealed that *S. flexneri* infection significantly elevated the inflammatory cell populations. Specifically, the populations of M1-type macrophages (CD86^+^, gated on CD11b^+^ F4/80^+^), Th1 cells (CD4^+^ IFN-γ^+^, gated on CD3^+^), and Th17 cells (CD4^+^ IL-17A^+^, gated on CD3^+^) were all increased. Conversely, Treg cells (CD25^+^ Foxp3^+^, gated on CD3^+^ CD4^+^) showed a marked reduction. BBR-HD treatment effectively reduced the levels of M1 macrophages, Th1 cells, and Th17 cells (Figure 3A–C and Figure S1A–D). Concurrently, both BBR-LD and BBR-HD treatment upregulated the proportion of Treg cells (Figure 3D and Figure S1E). To further validate this mechanism, we examined the mRNA levels of lineage-specific transcription factors in colonic tissues using RT-qPCR, including T-bet for Th1 cells, Ror-γt for Th17 cells, and Foxp3 for Treg cells. We found that the mRNA levels of *T-bet* and *Ror-γt* were significantly lower in BBR-treated mice than in the MO group. Additionally, BBR-HD treatment alleviated the suppressive effect of infection on *Foxp3*, leading to its upregulation. These findings indicated that BBR might modulate the expression of key transcription factors governing T cell differentiation, thereby rebalancing the Th1/Th17/Treg axis and restoring intestinal immune regulation during *S. flexneri*-induced intestinal inflammation (Figure 3E).

Th1 and Th17 cells drive inflammatory responses through the secretion of IFN-γ and IL-17A, respectively, while Treg cells maintain immune regulation by releasing IL-10 and TGF-β1. To determine whether BBR directly modulated these associated cytokine profiles, we quantified their levels in serum. As shown in Figure 3F, BBR treatment induced a clear anti-inflammatory shift: serum levels of IFN-γ and IL-17A were markedly decreased, while IL-10 and TGF-β1 were significantly elevated in BBR-treated mice compared to the MO group.

Additionally, we assessed the levels of sIgA, a critical immunoglobulin secreted into the gut lumen to maintain mucosal homeostasis. We found that BBR treatment significantly increased the level of sIgA in the colon (Figure 3G).

To further validate the direct effects of BBR on T cell differentiation, we cultured purified naïve CD4^+^ T cells from MLNs of *S. flexneri*-infected mice in vitro under Th1, Th17, or Treg polarizing conditions, in the presence or absence of BBR. The experimental workflow is illustrated in Figure 3H. As revealed in Figure 3I–K (Figure S1F–H), BBR treatment led to an obvious decrease in the percentages of Th1 cells (CD4^+^ IFN-γ^+^, gated on CD3^+^) and Th17 cells (CD4^+^ IL-17A^+^, gated on CD3^+^). In contrast, the percentage of Treg cells (CD25^+^ Foxp3^+^, gated on CD3^+^ CD4^+^) was markedly increased. Collectively, these results demonstrated that BBR exerted immunomodulatory effects by directly regulating the differentiation of Th1, Th17, and Treg cells in vitro, which is consistent with our in vivo findings.

## 3. Discussion

BBR has been used clinically for treating dysentery for many years. However, the precise mechanisms of action still remain incompletely elucidated. While BBR exhibits only modest direct bactericidal activity in vitro, its significant in vivo efficacy is well-established. This suggests that BBR’s protective effects are mediated primarily through the modulation of host-directed pathways. Our previous findings demonstrated that BBR resolved intestinal inflammation in a mouse model of *S. flexneri*-induced dysentery by inhibiting caspase-1-dependent macrophage pyroptosis and subsequent mitochondrial damage [12]. These findings prompted us to further investigate BBR’s role in intestinal immunity. Herein, we found that BBR protected against *S. flexneri*-induced dysentery in mice through enhancing intestinal barrier function and rebalancing inflammatory responses.

BBR has gained significant attention for its potent immunomodulatory properties. Its anti-tumor activity is partly attributed to its ability to accumulate within the tumor microenvironment, where it directly modulates T cell function. In non-small cell lung cancer, BBR has been shown to exert anti-tumor effects by downregulating PD-L1 expression while enhancing the immune activity of tumor-infiltrating T cells [19]. Similarly, BBR can attenuate rheumatoid arthritis (RA) through inducing apoptosis in mature dendritic cells (DCs) in spleens and lymph nodes to prevent hyperactivation of the immune system [20]. Moreover, BBR exhibits potential as an adjuvant for novel vaccines. Priming of CD8^+^ T cells with BBR led to improved central memory formation, primarily mediated by activation of AMPK and Jak3/Stat5. In vivo, mice vaccinated while on a BBR-supplemented diet conferred enhanced memory protection against infection [21]. In this study, we demonstrated that BBR alleviated *S. flexneri*-induced dysentery by attenuating M1 macrophage polarization and restoring the Th1/Th17/Treg balance, thereby rebalanced the dysregulated inflammatory responses. The detailed immune mechanisms involved need further investigation.

In addition to its clinical use in treating dysentery, BBR has also shown as a promising therapeutic agent for inflammatory bowel disease (IBD), a chronic and progressive intestinal inflammatory disease that includes Crohn’s disease (CD) and ulcerative colitis (UC) [22]. A key mechanism for BBR’s therapeutic effect is the enhancement of intestinal barrier. BBR can ameliorate DSS-induced UC in mice by preserving the intestinal barrier integrity through the upregulation of ZO-1 and E-cadherin proteins [23]. In addition, BBR exerts potent anti-inflammation effects. It can disrupt macrophage–epithelial interaction in a mouse intestinal organoid–macrophage co-culture system by inhibiting inflammatory chemokines to alleviate intestinal inflammation [24]. BBR has also been found to antagonize dopamine receptor-mediated signaling by suppressing secretion of inflammatory cytokines and modulating adaptive inflammatory responses in immune cells to ameliorate IBD [25]. Furthermore, BBR’s protective effects extend to colitis-associated cancer (CAC), where it functions through a microbiota-centered mechanism. BBR remodeled the gut microbiota, regulated short-chain fatty acid production, and inhibited the TLR4/NF-κB P65/IL-6/p-STAT3 inflammatory–cancer transformation pathway, thereby alleviating the CAC tumorigenesis [26]. Collectively, these studies have provided a comprehensive mechanistic framework of BBR in treating IBD, which can guide further investigation into the mechanisms for treating dysentery.

BBR is clinically recommended for the treatment of dysentery. For adult bacillary dysentery, the recommended dose is 100–300 mg, administered three times daily. Given an average human body weight of 70 kg, this corresponds to a dose range of 4.29–12.86 mg/kg/d. According to the conversion formula of body surface area to adjust dosages between mice and human, the equivalent dose range for mice is calculated to be 52.71–158.14 mg/kg/d. Therefore, the high-dose BBR (150 mg/kg/day) administered to mice in this study falls within this clinically equivalent range, ensuring its consistency with clinical usage and supporting the safety and translational relevance of our findings.

Based on our previous and current study, we have identified that BBR exerted its therapeutic effect on dysentery through a dual mechanism involving both bacterial and host targeting [12]. In the early stage of infection, the direct bactericidal effect of BBR rapidly reduces the pathogen load. As the infection and the tissue damage induced by inflammation increases, BBR alleviates the intestinal inflammation by maintaining barrier integrity and rebalancing the immune responses. Although the immunomodulatory effect of BBR is not directly associated with bacterial clearance, it facilitates the host immune recovery by establishing the balance of immune cells, thereby assisting in the clearance of residual pathogens. This synergy explains the superior effect of BBR in vivo than in vitro.

In summary, this study is the first to systematically elucidate the therapeutic mechanisms of BBR on intestinal barrier function and inflammatory responses in an *S. flexneri*-induced dysentery mouse model. Specifically, we revealed the intestinal protective mechanisms underlying BBR’s therapeutic effects against this infection, which are complementary evidence to the host-directed antibacterial effect of BBR. Collectively, these findings provide a solid theoretical basis for the clinical application of BBR in treating dysentery.

## 4. Materials and Methods

### 4.1. Chemicals and Antibodies

Berberine chloride was obtained from MedChem Express (MCE, Monmouth Junction, NJ, USA). Dulbecco’s Phosphate-Buffered Saline (DPBS), cell culture media, Penicillin-Streptomycin, and fetal bovine serum (FBS) were obtained from Thermo Fisher Scientific (Waltham, MA, USA). A fixation/permeabilization solution kit (BD GolgiPlug; BD Biosciences, Franklin Lakes, NJ, USA) and F4/80, CD11b, CD86, IFN-γ, CD25, Foxp3, CD4, and IL-17A antibodies used for macrophage, Th1, Th17, and Treg cell phenotyping were purchased from BD Biosciences (Franklin Lakes, NJ, USA).

### 4.2. Animal Infections

Female C57BL/6J mice (5 weeks old, 20 ± 2 g) were obtained from SiPeiFu Biotechnology (Nanjing, China). Mice were housed in a temperature- (22 ± 1 °C), humidity- (55 ± 20%), and light-controlled (12 h light/dark cycle) environment. The mice were acclimatized for 7 days before infection with *S. flexneri*.

The experimental protocol followed established laboratory methods [12]. Briefly, the in vivo study was conducted as a single, independent experiment. Mice were randomly divided into four groups of 10 animals each: normal control group (NC), model group (MO), low-dose BBR-treated model group (BBR-LD) (50 mg/kg/d), and high-dose BBR-treated model group (BBR-HD) (150 mg/kg/d). BBR was orally administered for 7 days pre-infection. Mice were then intraperitoneally (i.p.) challenged with 2.5 × 10^8^ CFU *S. flexneri*. The burden of *S. flexneri* in colonic tissues and whole blood were assessed by SS agar plating at 8 h post-infection, with colon length measured concurrently. Mice were anesthetized with an i.p. injection of 1% pentobarbital sodium, and approximately 100 μL of whole blood was collected into sterile tubes via retro-orbital puncture using a glass capillary tube. Mice were euthanized by cervical dislocation immediately after blood collection. Serum was separated by allowing the blood to clot at room temperature for 30 min, followed by centrifugation at 3000× *g* for 10 min.

### 4.3. Disease Score

Mice from all experimental groups were monitored daily based on weight loss, stool consistency, and general appearance. Each score was determined as follows: change in body weight loss (0: ≤1%; 1: 1–5%; 2: 5–10%; 3: 10–15% and 4: ≥15%), stool consistency (0: normal, 1: semiloose stool (^+^), 2: mild diarrhea (^++^), 3: liquid stool (^+++^), 4: liquid stool (^++++^)) and general appearance (0: normal, 1: piloerection, 2: lethargy and piloerection, 4: motionless and sickly). The sum of these scores constituted the disease score.

### 4.4. Histological Analysis

Colon tissues were fixed in 4% paraformaldehyde and then embedded in paraffin. Subsequently, 5 μm sections were stained with hematoxylin and eosin (H&E) (Servicebio, Wuhan, China). The epithelial scoring criteria are as follows: 0, normal morphology; 1, loss of goblet cells; 2, loss of goblet cells in large areas; 3, loss of crypts; 4, loss of crypts in large areas. The inflammatory infiltration scoring criteria are as follows: 0, no infiltrate; 1, infiltrate around crypts; 2, infiltrate reaching the lamina muscularis mucosae; 3, extensive infiltration reaching the lamina muscularis mucosae and thickening of the mucosa; 4, infiltration of the submucosal layer. A total H&E score equals the sum of both scores.

### 4.5. Western Blot Analysis

Colon tissues were homogenized in RIPA buffer supplemented with protease and phosphatase inhibitors. Protein concentrations were determined using a BCA protein assay kit (Thermo Fisher Scientific). Equal amounts of protein were resolved by 10% SDS-PAGE and subsequently transferred to PVDF membranes (Millipore, Burlington, MA, USA). After blocking with 5% NON-Fat milk for 1 h at room temperature, membranes were incubated with primary antibodies against ZO-1 (1:1000, CST13663, Cell Signaling Technology, Danvers, MA, USA), E-cadherin (1:1000, CST3195), occludin (1:1000, CST91131), and β-actin (1:1000, CST8457) overnight at 4 °C. Membranes were then incubated with HRP conjugated secondary antibodies (1:5000, CST7074) for 2 h at room temperature. The protein bands were visualized by ChemiDoc XRS imaging system (Bio-Rad Laboratories, Hercules, CA, USA).

### 4.6. Immunofluorescence

Colon sections were fixed, permeabilized, blocked, and then incubated with ZO-1 (1:1000, CST13663), E-cadherin (1:1000, CST3195), and occludin (1:1000, CST91131) primary antibodies overnight at 4 °C. And then the nucleus was stained with DAPI (ab285390, Abcam, Cambridge, MA, USA). Images were collected on Leica TCS SPS microscope (Wetzlar, Germany).

### 4.7. Flow Cytometry Analysis

Mouse mesenteric lymph nodes (MLNs) were harvested and gently pressed through a 70 μm nylon mesh strainer in precooled DPBS to obtain a single cell suspension (1 × 10^6^ cells/mL). For Th1 and Th17 cell intracellular staining, single cells were prestimulated using GolgiPlug for 6 h at 37 °C at a concentration of 2 μL/mL. Then, (CD11b^+^, F4/80^+^ and CD86^+^) M1 macrophages, (CD4^+^, IFN-γ^+^) Th1, (CD4^+^, IL-17A^+^) Th17, and (CD4^+^, CD25^+^ and Foxp3^+^) Treg lymphocytes were labeled separately using FACS antibodies according to the instruction manual and analyzed by FACS Verse (BD Biosciences). The data were analyzed using FlowJo 10 software (TreeStar, San Carlos, CA, USA).

### 4.8. Enzyme-Linked Immunosorbent Assay (ELISA)

Cytokines concentrations in serum were measured using mouse sIgA, IFN-γ, IL-17A, IL-10, and TGF-β1 ELISA kits (Elabscience, Wuhan, China) according to the manufacturer’s instructions.

### 4.9. RNA Extraction and Real-Time Quantitative Polymerase Chain Reaction (RT-qPCR)

Total RNA was extracted from colon tissues using TRIzol Plus RNA Purification Kit (Invitrogen, Waltham, MA, USA) and then reverse transcribed by HiFiScript cDNA Synthesis Kit for qPCR (CWBIO, Beijing, China). Real-time PCR was performed with UltraSYBR Mixture (Low ROX) (CWBIO, Beijing, China) on an Applied Biosystems 7500 Fast Real-Time PCR System (Applied Biosystems, Foster City, CA, USA). The primers used for PCR amplification are listed in Supplementary Materials Table S1. The fold changes in mRNA expressions were normalized to Gapdh using the ΔΔ Ct method.

### 4.10. Isolation of Naïve CD4^+^ T Cells and Th Cell Differentiation

Naïve CD4^+^ T cells were isolated from MLN cells of infected mice using the EasySep™ mouse naïve CD4^+^ T cell isolation kit (Stem cell, Vancouver, BC, Canada) according to the instructions. The purified naïve CD4^+^ T cells were then stimulated with plate-bound anti-CD3 (5 μg/mL) and soluble anti-CD28 (3 μg/mL) under specific polarizing conditions for Th1, Th17, and Treg differentiation in the presence or absence of BBR (0, 12.5, 25, or 50 μM). Th1 polarizing conditions are 5 μg/mL anti-IL-4, 15 ng/mL IL-12, and 40 ng/mL IL-2. Th17 polarizing conditions are 5 μg/mL anti-IL-4, 5 μg/mL anti-IFN-γ, 3 ng/mL TGF-β, 20 ng/mL IL-6, and 40 ng/mL IL-2. Treg polarizing conditions are 5 μg/mL anti-IL-4, 5 μg/mL anti-IFN-γ, 15 ng/mL TGF-β, and 40 ng/mL IL-2. After 4–5 days activation, all cells were collected for FACS analysis.

### 4.11. Statistical Analysis

Data were processed by GraphPad Prism 9.0 software (GraphPad Software, La Jolla, CA, USA) and are presented as mean ± SD. One-way ANOVA followed by Tukey’s post hoc test was used to compare the multiple groups to evaluate the statistically significant variance. *p*-values of less than 0.05 were considered statistically significant.

## Data Availability

The original contributions presented in this study are included in the article/Supplementary Materials. Further inquiries can be directed to the corresponding author.

## Supplementary Materials

The following supporting information can be downloaded at https://www.mdpi.com/article/10.3390/ijms27021063/s1.

## Abbreviations

The following abbreviations are used in this manuscript:

BBR	Berberine
sIgA	secretory immunoglobulin A
MIC	Minimal inhibitory concentration
MLNs	Mesenteric lymph nodes
*S. flexneri*	*Shigella flexneri*
