# Supplementary material for: Berberine Alleviates Shigella-Induced Dysentery by Regulating Intestinal Barrier and Inflammatory Responses
Source: Int J Mol Sci. 2026 Jan 21;27(2):1063. doi: 10.3390/ijms27021063 (PMC12842174; doi:10.3390/ijms27021063)
Supplement: Supplementary file 1 [file ijms-27-01063-s001.zip › ijms-4077148-supplementary.pdf]

Supplementary Materials

Table S1. Primer sequence for quantitative PCR.

Gene	Forward Primer (from 5' to 3')	Reverse Primer (from 5' to 3')
Gapdh	CTCCCACTCTTCCACCTTCG	TAGGGCCTCTCTTGCTCAGT
Il-17a	AGACTACCTCAACCGTTCCA	CTCGACCCTGAAAGTGAAGG
Tgf-β1	CTGATACGCCTGAGTGGCTG	TTTGGGGCTGATCCCGTTG
Il-10	TACGTGGCTGTTACATGTAGGG	GATTTGTCCGTCTGCTTTGGG
Iga	GGGAAGGTACCAAGAACCGC	GTCATCTGGCATGTCCACCC
Ifn-γ	ACTGGCAAAAGGATGGTGACA	GACTCCTTTTCCGCTTCCTGA
T-bet	CGCTTATATGTCCACCCAGAC	GAGAGACTGCAGGACGATCA
Ror-γt	AATGTCTGCAAGTCCTTCCG	CTCCCACATCTCCCACATTG
Foxp3	GGGGAAGCCATGGCAATAGT	GCGGGGTGGTTTCTGAAGTA

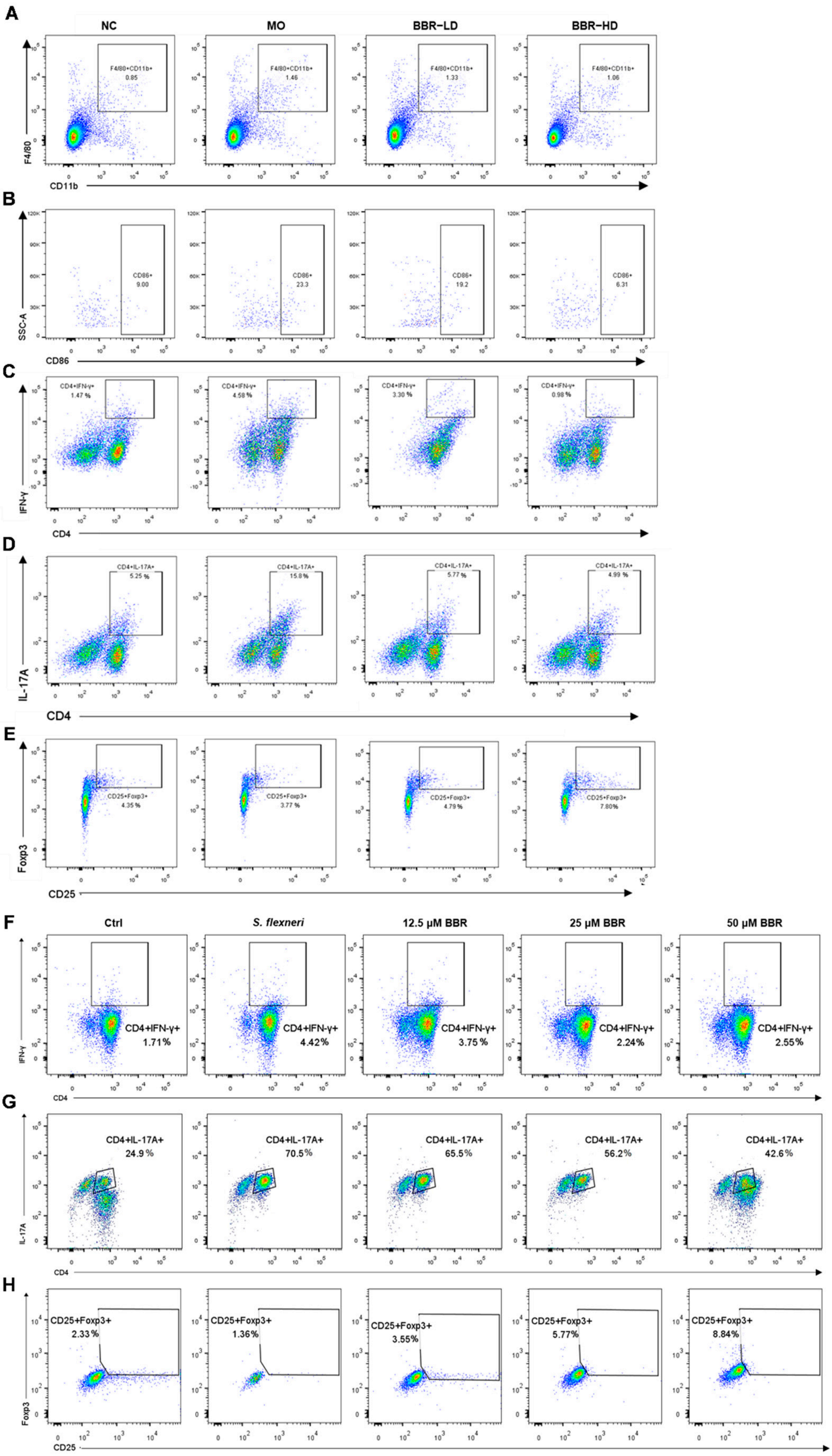

Figure S1. Effect of BBR on the proportion of immune cells. (**A**,**B**) Flow cytometry analysis of macrophages (**A**) and M1-like macrophages (**B**) in the MLN samples of mice. (**C**–**E**) Flow cytometry analysis of Th1 (**C**), Th17 (**D**) and Treg (**E**) cells in the MLN samples of mice. (**F**–**H**) Flow cytometry analysis of naïve CD4^{+} T cells polarization into Th1 (**F**), Th17 (**G**) and Treg (**H**) cells.
